# Supplementary figures and images for: C/VDdb: A multi-omics expression profiling database for a knowledge-driven approach in cardiovascular disease (CVD)
Source: PLoS One. 2018 Nov 12;13(11):e0207371. doi: 10.1371/journal.pone.0207371 (PMC6231654; doi:10.1371/journal.pone.0207371)

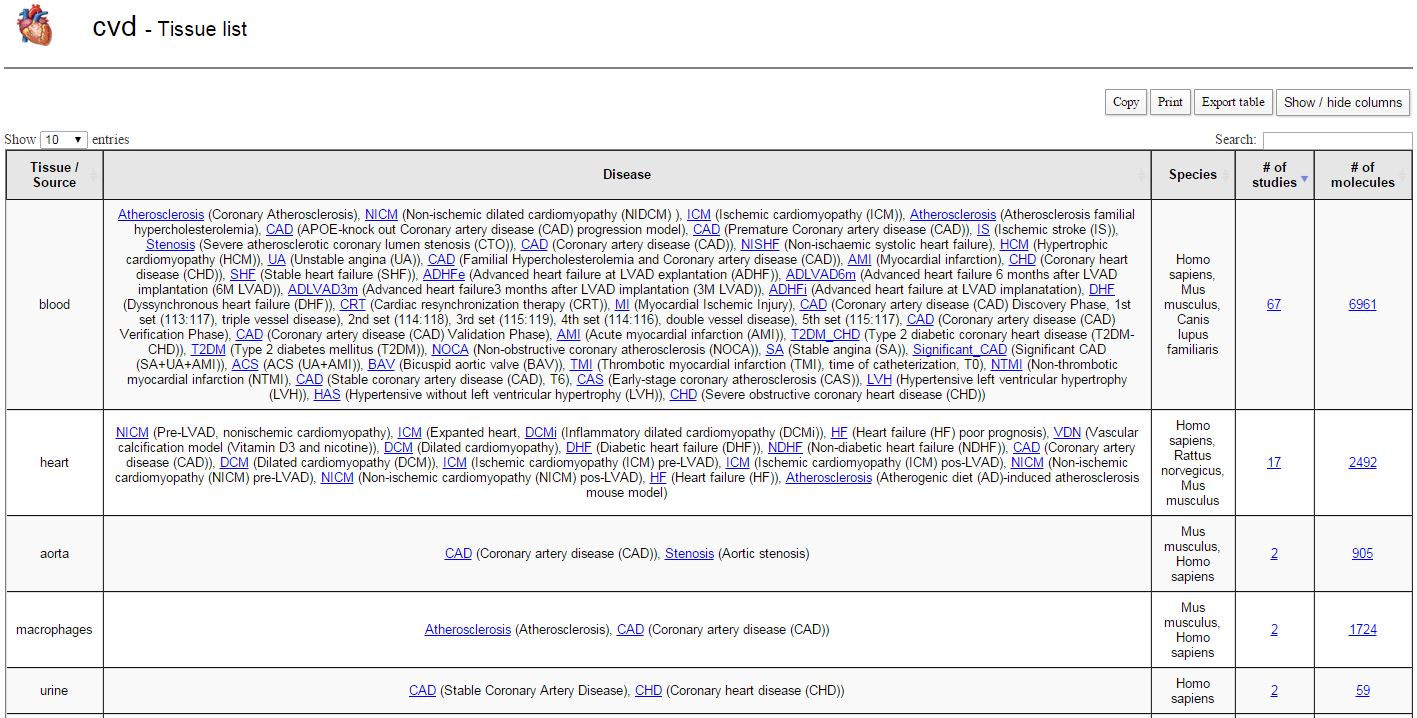

Supplement: S1 Fig — The fields of disease, species, number of studies and number of molecules per each tissue/fluid (blood, heart, aorta, urine) source are described. (TIF) [file pone.0207371.s001.tif]

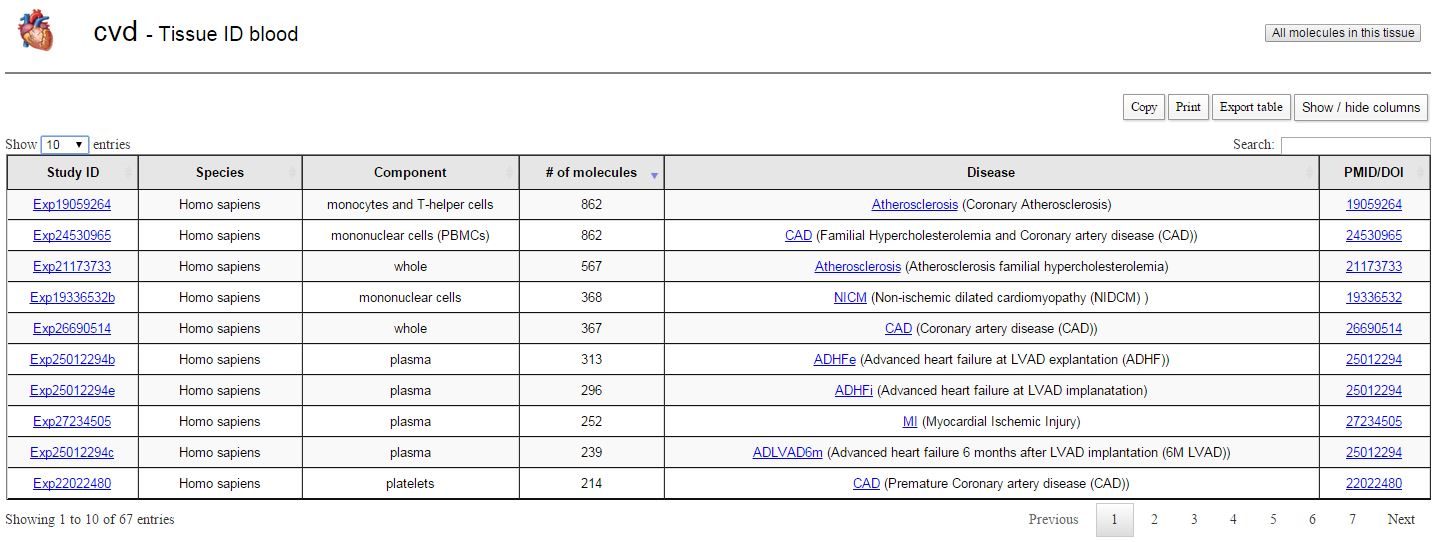

Supplement: S2 Fig — The fields of species, component, number of molecules, disease, and PMID/DOI are described per study. (TIF) [file pone.0207371.s002.tif]

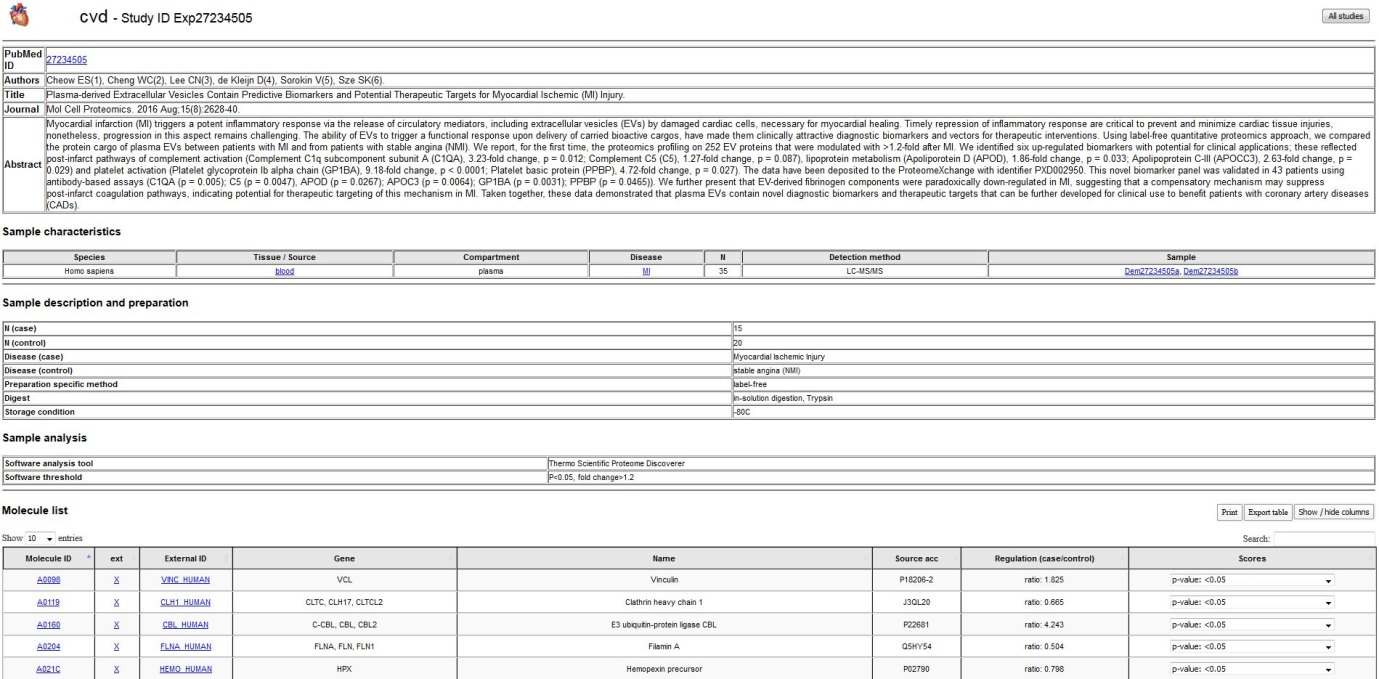

Supplement: S3 Fig — First headers enclose the main bibliometric data associated with a research article. The second and third headers are with respect to the description of the sample itself and several steps in their processing till reach molecule identification. The fourth header corresponds to the incoming molecules from the previous step. It lists all the molecules identified in the study with the associated statistical scores, fold-change and regulation. (TIF) [file pone.0207371.s003.tif]

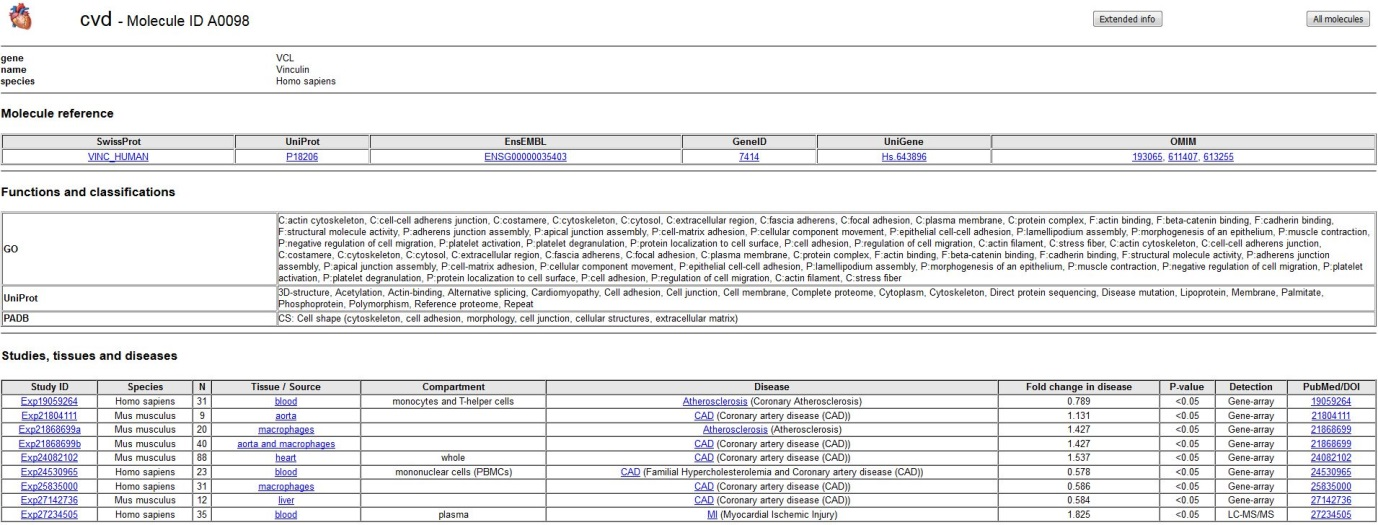

Supplement: S4 Fig — In the first header is displayed the linkage to external databases. Following it we have represented the associated function and classification and as well our own type of tag for functional classification (derived from PADB). The third header corresponds to the number of occurrences of the molecule across all the studies, disease conditions, species, and tissue/fluid source. (TIF) [file pone.0207371.s004.tif]

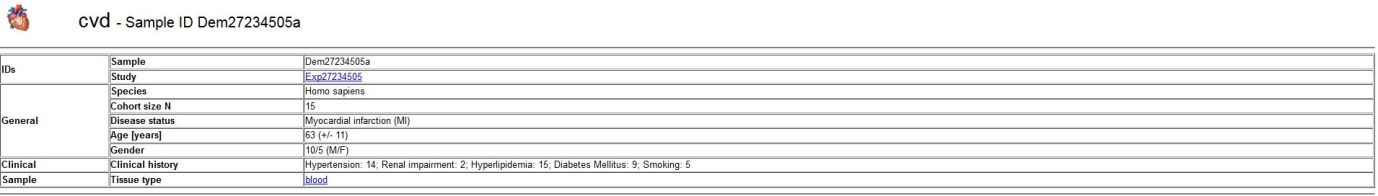

Supplement: S5 Fig — Clinical data (demographics page view) is also represented in the C/VD database and is linked with the study page and vice-versa. (TIF) [file pone.0207371.s005.tif]

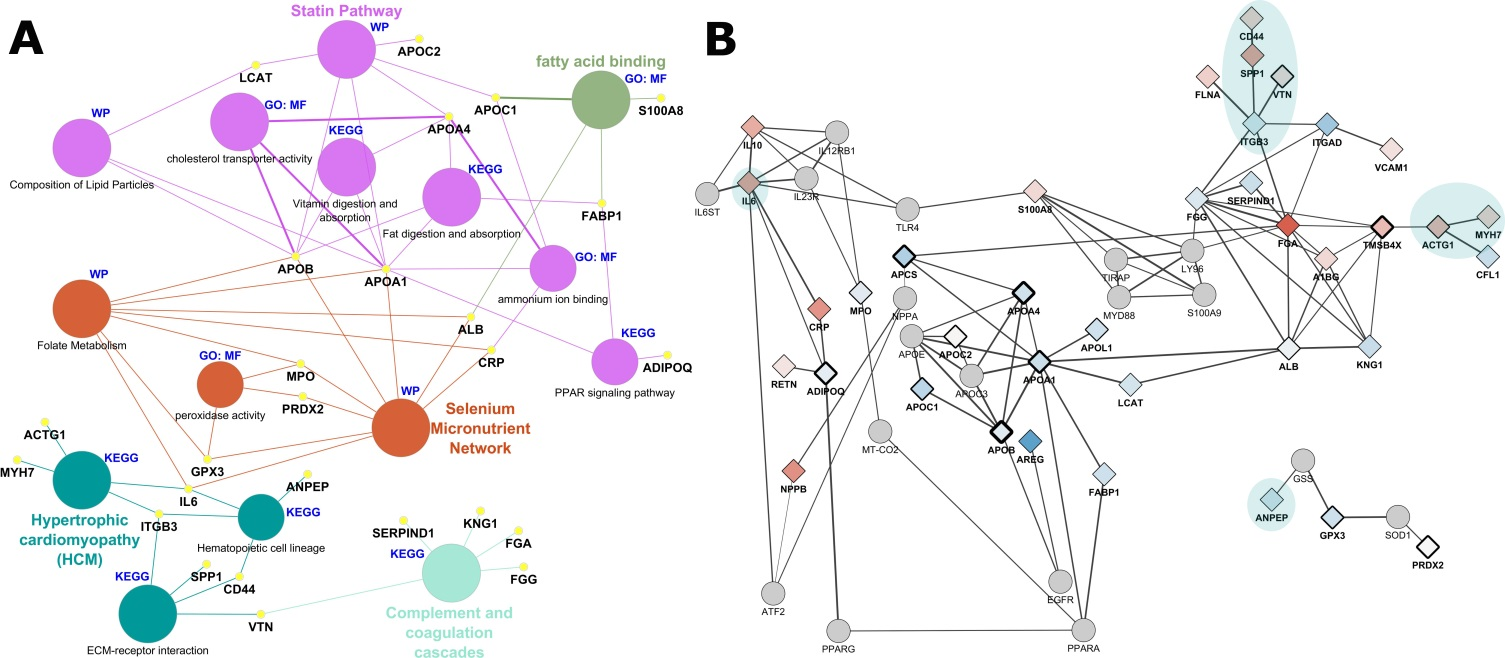

Supplement: S6 Fig — A: Gene ontology (GO) analysis. B: STRING protein-protein interactions with a minimum confidence score of 0.70. The light blue demarked oval in B denotes proteins from the hypertrophic cardiomyopathy (HCM) and ECM-receptor interactions ClueGO derived cluster. WikiPathways (WP), biological process (BP), molecular function (MF) and cell component (CC). (TIF) [file pone.0207371.s006.tif]

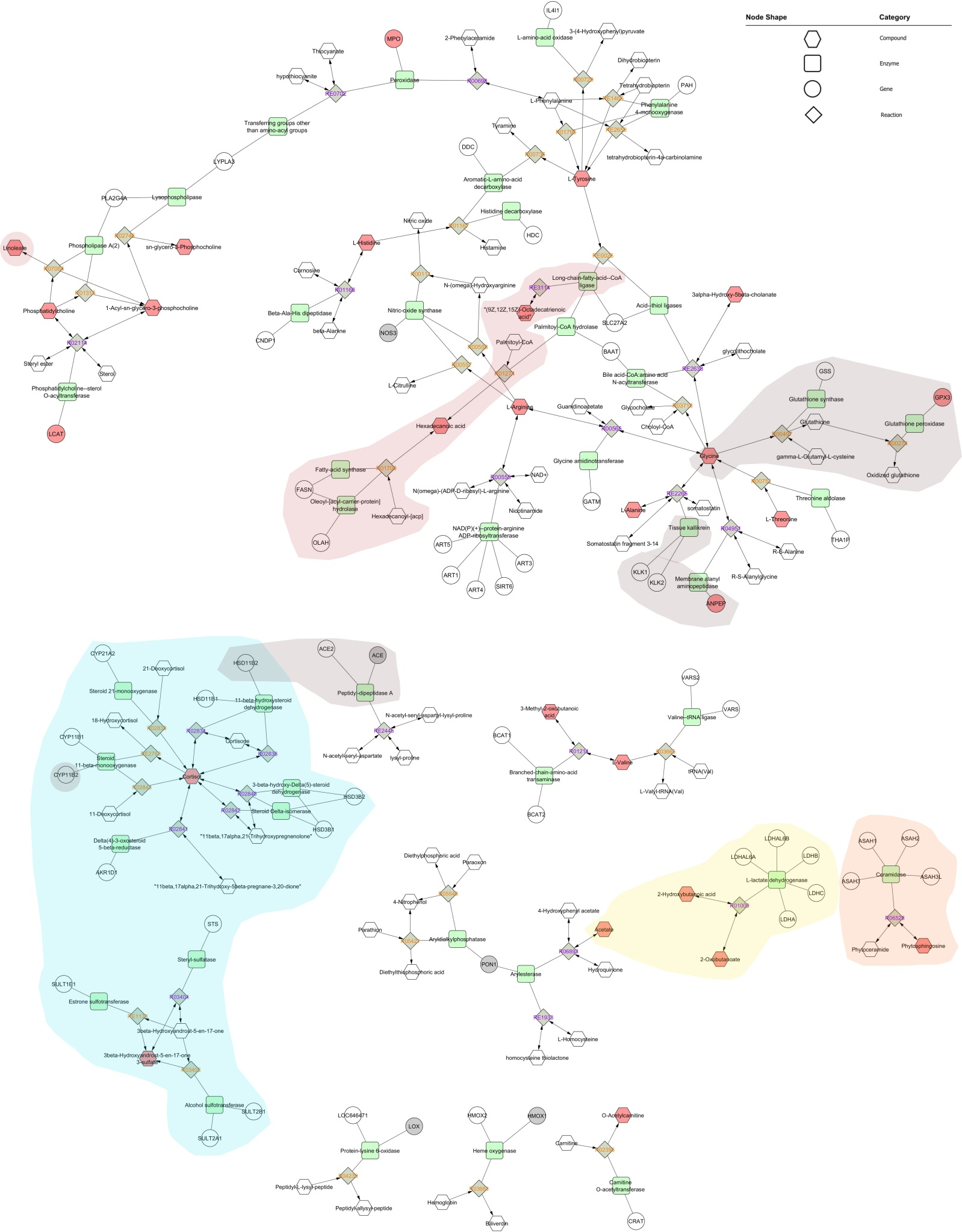

Supplement: S7 Fig — Matched compounds and gene nodes are pink colour filled when derived from the C/VD database and grey when from DisGeNET regarding coronary artery disease (CAD) association. (TIF) [file pone.0207371.s007.tif]

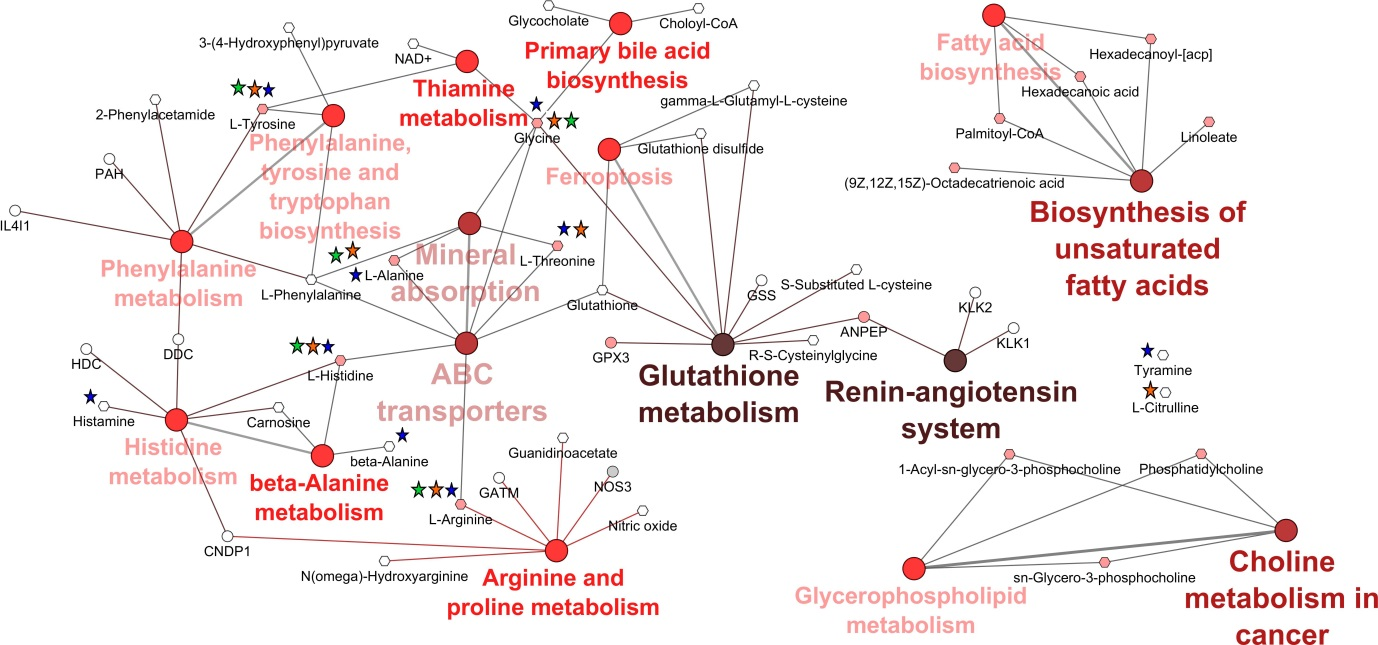

Supplement: S8 Fig — Most significant enriched node terms are displayed darker. Colour filled compounds and proteins/genes are from CAD datasets (exception for NOS3 that is derived from DisGeNET). To improve network visualisation some ontology terms were removed from the figure, such as protein digestion and absorption: blue stars; biosynthesis of amino acids: orange stars; central carbon metabolism in cancer: green stars. (TIF) [file pone.0207371.s008.tif]

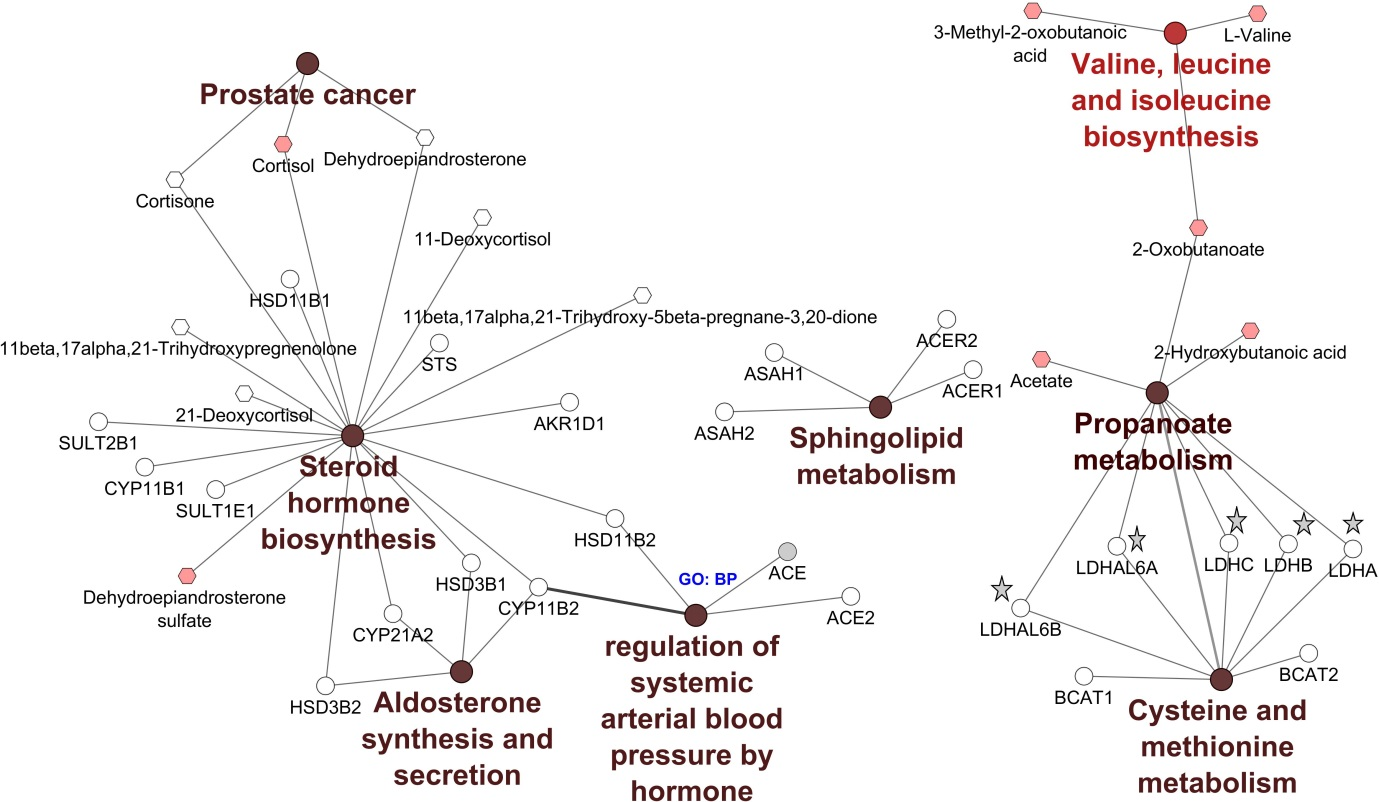

Supplement: S9 Fig — Most significant enriched node terms are displayed darker. Colour filled compounds and proteins/genes are from CAD datasets (exception for ACE that is derived from DisGeNET). To improve network visualisation some ontology terms were removed from the figure, such as pyruvate metabolism, glucagon signaling pathway, and glycolysis/gluconeogenesis that have a grey start annotation. (TIF) [file pone.0207371.s009.tif]

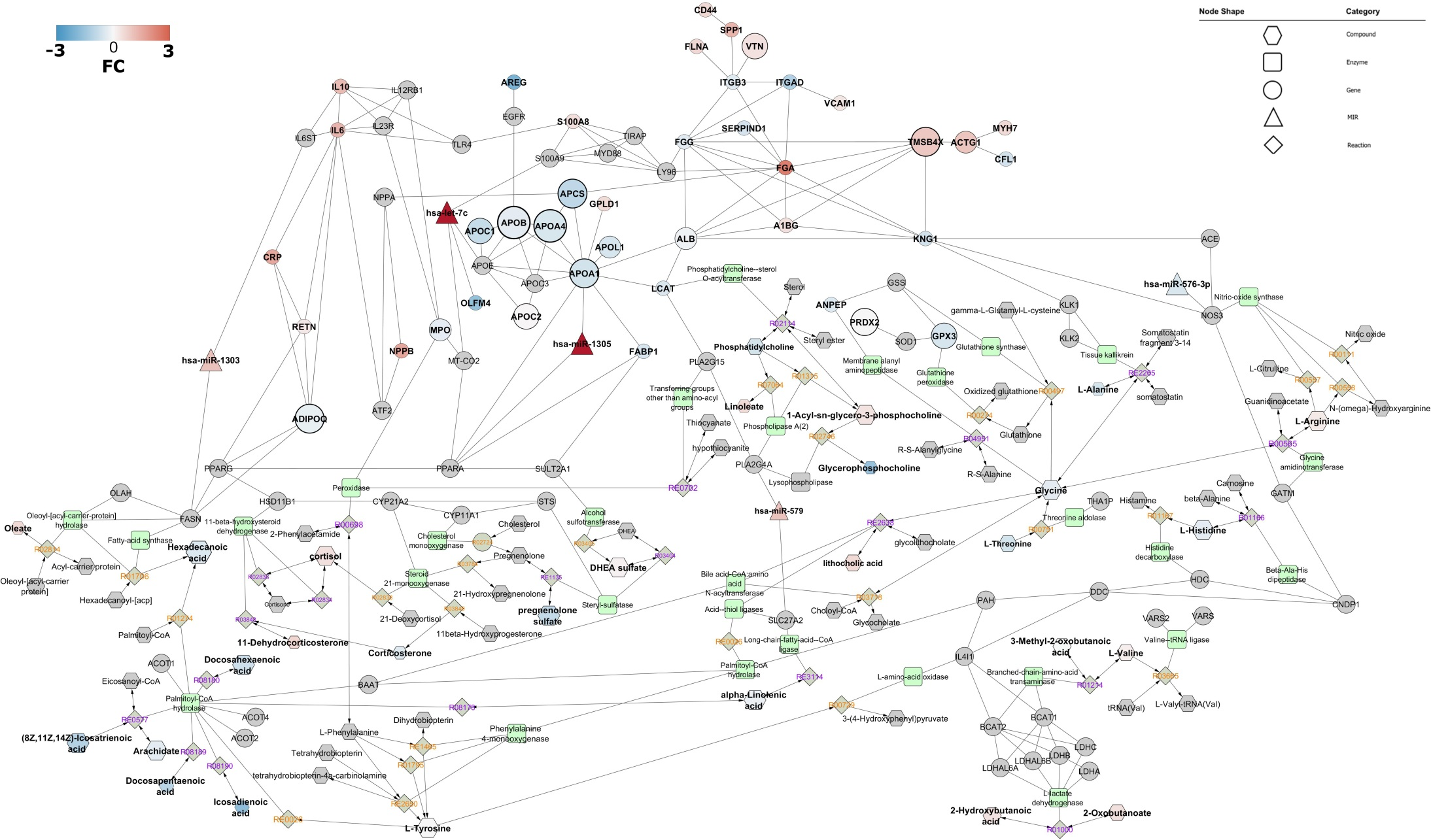

Supplement: S10 Fig — Network representation of microRNAs as node triangles, proteins/genes as circles, enzymes displayed as round rectangles, compounds as hexagons, and reactions shaped as diamonds, labels colour coded as irreversible/directed: orange, reversible/bidirected: purple. Enrichment using protein-protein physical interactions is derived from STRING with an established minimum confidence score of 0.70. Gene-enzyme-reaction-compound associations were established using MetScape 3.1.3 and KEGG. MicroRNA-targets associations were derived using miRanda and TarBase. Differential expression is represented as a colour gradation from blue (decreased expression) to red (increased expression). Node size is proportional to the number of molecules reported within data sets. (TIF) [file pone.0207371.s010.tif]
