# Supplementary material for: C/VDdb: A multi-omics expression profiling database for a knowledge-driven approach in cardiovascular disease (CVD)
Source: PLoS One. 2018 Nov 12;13(11):e0207371. doi: 10.1371/journal.pone.0207371 (PMC6231654; doi:10.1371/journal.pone.0207371)
Supplement: S1 File — (DOCX) [file pone.0207371.s011.docx]

**S1 File. Supplementary material.**

S1 Table. Clinical parameters of the human cohorts used in the CAD studies. high-density lipoprotein (HDL); blood pressure (BP); Body-mass-index (BMI); Males (M), Females (F); coronary artery disease (CAD), coronary disease (CD), acute myocardial infarction (AMI), stable angina (SA), unstable angina (UA), Non-obstructive coronary atherosclerosis (NOCA), normal coronary artery (NCA).

| **EXPREF** | **DEMOGRAPHREF** | **age (case\|control)** | **gender (case\|control)** | **clinical history case** | **clinical history control** |
| --- | --- | --- | --- | --- | --- |
| Exp22022480 | Dem22022480a\|Dem22022480b | 45.7+/-6.5\|45.4+/-7.0 | 126/78\|81/69(M/F) | CAD defined as AMI. n (%): 6 (50); CAD defined as stable angina. n (%): 6 (50) |  |
| Exp23216991 | Dem23216991a\|Dem23216991b | 62.8+/-10.6\|57.9+/-10.5 |  | HDL: 41.6+/-9.9; EF (%): 53.1+/-10.9; Diabetes: 31; BMI: 29.8+/-5.1 | < 50% obstruction in any epicardial vessel, insignificant coronary artery disease; HDL: 50.5+/-18.2; EF (%): 57.8+/-8.4; Diabetes: 16; BMI: 30.6+/-7.6 |
| Exp24859250 | Dem24859250a\|Dem24859250b | 57.76\|56.46 | 10/0\|10/0(M/F) | Diabetes: 0; Hypertension: 4; Antidiabetic agents: 0; Antihypertensive agents: 4; Aspirin: 4; BMI: 26.06 | Diabetes: 0; Hypertension: 4; Antidiabetic agents: 0; Antihypertensive agents: 4; Aspirin: 4; BMI: 25.56 |
| Exp25790721 | Dem25790721a\|Dem25790721b | 51.4+/-12.4\|55.89+/-2.94 |  | Smoking-no. (%): 7 (36.8); Diet-veg. (%): 9 (47.4); Hypertension status-no. (%): 4 (21.1); Diabetes status-no. (%): 5 (26.3); BMI: 23.3 | Smoking-no. (%): 6 (31.6); Diet-veg. (%): 8 (42.1); Hypertension status-no. (%): 4 (21.1); Diabetes status-no. (%): 2 (10.5); BMI: 26.96 |
| Exp26932197a | Dem26932197a\|Dem26932197b | 35-79\|50-70 |  |  |  |
| Exp27350024a1\|Exp27350024a2\|Exp27350024a3\|Exp27350024a4\|Exp27350024a5 | Dem27350024a\|Dem27350024b | 56.25+/-5.24\|58.17+/-4.21 |  | Hypertension (2), Diabetes Mellitus (2); BMI: 22.0-24.85 | Hypertension (2), Diabetes Mellitus (2); BMI: 21.76-27.11 |
| Exp27350024b | Dem27350024c\|Dem27350024d | 50.05+/-2.03\|52.55+/-5.10 |  | Hypertension (4), Diabetes Mellitus (6); BMI: 23.3-28.28 | Hypertension (1), Diabetes Mellitus (2); BMI: 22.43-28.8 |
| Exp27350024c | Dem27350024e\|Dem27350024f | 51.74+/-8.75\|48.24+/-10.36 |  | Hypertension (41.1), Diabetes Mellitus (30.8); BMI: 22.58-27.43 | Hypertension (15), Diabetes Mellitus (11); BMI: 22.57-26.52 |
| Exp27394176a | Dem27394176a\|Dem27394176b | 68.8+/-9.3\|63.0+/-14.1 |  | BMI: 23.8+/-2.7; systolic BP (mmHg): 133.7+/-26.2; diastolic BP (mmHg): 71.6+/-13.9 | BMI: 22.0+/-2.8; systolic BP (mmHg): 117.1+/-11.1; diastolic BP (mmHg): 72.9+/-8.8 |
| Exp27470195a | Dem27470195a\|Dem27470195c | 67.1+/-5.8\|65.3+/-6.6 | 15/13\|7/8(M/F) | BMI: 26.31+/-2.62; systolic BP (mmHg): 125.17+/-12.02; diastolic BP (mmHg): 76.83+/-11.13 | BMI: 25.63+/-2.04; systolic BP (mmHg): 121.75+/-10.65; diastolic BP (mmHg): 78.66+/-9.58 |
| Exp27634119a | Dem27634119a\|Dem27634119b | 61.6+/-8.3\|57.7+/-8.4 | 140/136\|62/54(M/F) | Hypertension (%): 69.2; Diabetes mellitus (%): 16.7; Current smoker (%): 18.5; BMI: 24.8+/-3.8; systolic BP (mmHg): 134.5+/-16.8; diastolic BP (mmHg): 82.9+/-11.0 | Hypertension (%): 55.2; Diabetes mellitus (%): 12.1; Current smoker (%): 19.8; BMI: 24.6+/-3.9; systolic BP (mmHg): 129.6+/-18.2; diastolic BP (mmHg): 81.5+/-11.0 |
| Exp27634119k | Dem27634119a\|Dem27634119b\|Dem27634119c\|Dem27634119d\|Dem27634119e | 64.6+/-8.2 (SA)\|65.0+/-9.2 (UA)\|63.6+/-10.8 9 (AMI)\|57.7+/-8.4\|61.6+/-8.3 (NOCA) |  | Hypertension (%): 85.7; Diabetes mellitus (%): 23.8; Current smoker (%): 15.9 (SA)\|Hypertension (%): 76.9; Diabetes mellitus (%): 29.3; Current smoker (%): 28 (UA)\|Hypertension (%): 71.1; Diabetes mellitus (%): 29.8; Current smoker (%): 33.9 (AMI); BMI: 25.4+/-3.4 (SA)\|24.7+/-3.1 (UA)\|24.3+/-3.3 (AMI); BP: systolic BP (mmHg): 141.6+/-18.7 ; diastolic BP (mmHg): 82.8+/-9.1 (SA)\|systolic BP (mmHg): 140.0+/-17.4 ; diastolic BP (mmHg): 81.5+/-10.2 (UA)\|systolic BP (mmHg): 133.6+/-20.7 ; diastolic BP (mmHg): 83.0+/-14.6 (AMI) | Hypertension (%): 55.2; Diabetes mellitus (%): 12.1; Current smoker (%): 19.8 (NCA)\|Hypertension (%): 69.2; Diabetes mellitus (%): 16.7; Current smoker (%): 18.5 (NOCA); BMI: 24.6+/-3.9 (NCA)\|24.8+/-3.8 (NOCA); BP: systolic BP (mmHg): 129.6+/-18.2 ; diastolic BP (mmHg): 81.5+/-11.0 (NCA)\|systolic BP (mmHg): 134.5+/-16.8 ; diastolic BP (mmHg): 82.9+/-11.0 (NOCA) |
| Exp28205634 |  |  |  |  |  |
| Exp28414761c | Dem28414761c | 64.5+/-9.2 | 8/6(M/F) | BMI: 33.8+/-6.6 |  |
| Exp28414761f | Dem28414761c | 64.5+/-9.2 | 8/6(M/F) | BMI: 33.8+/-6.6 |  |
| Exp28924163 | Dem28924163a\|Dem28924163b | 58.43+/-9.69\|55.88+/-9.50 | 37/23\|29/31\|(M/F) | Hypertension, no, (%): 27(45.00) Hyperlipidemia, no, (%): 7(11.67); Prior coronary artery disease, no, (%): 2(3.33); Ejection fraction (%): 65.49+/-7.24; Number of artery stenosis: 1.95+/-0.81; Percentage of stenosis (%): 36.53+/-12.82; weight (kg): 70.03+/-10.41 | Hypertension, no, (%): 19(31.67); Hyperlipidemia, no, (%): 4(6.67); Prior coronary artery disease, no, (%): 0(0.00); Ejection fraction (%): 65.51+/-6.60; Number of artery stenosis: 0; Percentage of stenosis (%): 0; weight (kg): 69.21+/-10.70 |
| Exp29127404 | Dem29127404a\|Dem29127404b | 55.24+/-10.16\|55.23+/-10.23 | 75/75\|75/75(M/F) | Diabetes, (%): 40.7; Hypertension, (%): 62.0; Hyperlipidaemia, (%): 81.3; Smokers, (%): 46.0; Family history of CAD, (%): 32.7; Single vessel disease, (%): 14.7; Double vessel disease, (%): 29.3; Triple vessel disease, (%): 56, BMI: 26.25+/-4.01 | Diabetes, (%): 12.7; Hypertension, (%): 51.3; Hyperlipidaemia, (%): 66.0; Smokers, (%): 33.3; Family history of CAD, (%): 20.0; Single vessel disease, (%): 0; Double vessel disease, (%): 0, Triple vessel disease, (%): 0; BMI: 26.24+/-4.18 |

**S2 Table.** Expression matrix of the most reported molecular entities in CAD. PRO: protein, MIR: miRNA, MET: metabolite. CluSO identifiers from PADB. Log2(fold-change) and p-value <0.05.

| Tag | CluSO | Exp1 | Exp2 | Exp3 | Exp4 | Exp5 | Exp6 | Exp7 | Reported |
| --- | --- | --- | --- | --- | --- | --- | --- | --- | --- |
| PRO | B0853 | 0.138 | -0.338 | -0.308 | -0.427 | -0.346 | -0.173 | -0.761 | 7 |
|  | B0851 | -0.461 | -0.277 | -0.535 | -0.421 | -1.092 | -0.322 |  | 6 |
|  | B0397 | -0.55 | 0.294 | -0.076 | -0.514 | -0.415 |  |  | 5 |
|  | BH432 | 1.187 | 0.62 | -0.321 | 1.283 | 2.001 |  |  | 5 |
|  | BE542 | -0.555 | -1.683 | -1.412 | -0.829 | -0.224 |  |  | 5 |
|  | BC830 | -0.131 | 0.352 | -0.183 | -0.106 |  |  |  | 4 |
|  | B0856 | -0.933 | 0.335 | 0.714 |  |  |  |  | 3 |
|  | BH950 | 0.518 | 0.365 | 0.447 |  |  |  |  | 3 |
|  | BS056 | -0.672 | -0.246 | -0.333 |  |  |  |  | 3 |
|  | B6740 | -1.087 | -0.286 | -0.262 |  |  |  |  | 3 |
|  | B0855 | -0.351 | -1.793 | -0.278 |  |  |  |  | 3 |
|  | B0556 | -0.123 | -0.184 |  |  |  |  |  | 2 |
|  | B0598 | 0.439 | 0.615 |  |  |  |  |  | 2 |
|  | B0849 | -0.943 | -0.646 |  |  |  |  |  | 2 |
|  | B0863 | -0.711 | -0.353 |  |  |  |  |  | 2 |
|  | B0303 | 0.693 | 1.307 |  |  |  |  |  | 2 |
|  | BC163 | -1.057 | 0.444 |  |  |  |  |  | 2 |
| MIR | BZ197 | 0.124 | -0.576 |  |  |  |  |  | 2 |
|  | BZO58 | -0.515 | 0.005 |  |  |  |  |  | 2 |
|  | BZ004 | 0.345 | 7.791 |  |  |  |  |  | 2 |
|  | BZ167 | 0.287 | 1.848 |  |  |  |  |  | 2 |
|  | BZ169 | 0.227 | 13.379 |  |  |  |  |  | 2 |
|  | BZI97 | -0.136 | 2.401 |  |  |  |  |  | 2 |
|  | BZP20 | 0.176 | 1.891 |  |  |  |  |  | 2 |
|  | BZT43 | -0.396 | 2.248 |  |  |  |  |  | 2 |
|  | BZM86 | 0.506 | -5.644 |  |  |  |  |  | 2 |
|  | BZ536 | 0.275 | -5.851 |  |  |  |  |  | 2 |
|  | BZN57 | -0.152 | -1.089 |  |  |  |  |  | 2 |
|  | BZD94 | 0.516 | -1.515 |  |  |  |  |  | 2 |
|  | BZM84 | -0.396 | -2.12 |  |  |  |  |  | 2 |
|  | BZO69 | -0.415 | -4.322 |  |  |  |  |  | 2 |
|  | BZE90 | -0.152 | -2.184 |  |  |  |  |  | 2 |
|  | BZF39 | -0.152 | -1.029 |  |  |  |  |  | 2 |
|  | BZM93 | 0.516 | -1.515 |  |  |  |  |  | 2 |
|  | BZN59 | 0.189 | -1.599 |  |  |  |  |  | 2 |
|  | BZQ76 | 0.098 | -4.644 |  |  |  |  |  | 2 |
| MET | Z5E3C | -0.103 | -0.407 | 0.168 | -0.585 |  |  |  | 4 |
|  | Z401N | -0.396 | -0.065 | -0.246 | -0.227 |  |  |  | 4 |
|  | Z401G | -0.358 | -0.585 | -0.591 | -0.383 |  |  |  | 4 |
|  | Z401I | -0.569 | -0.381 | 3.184 | -0.585 |  |  |  | 4 |
|  | Z401U | -0.737 | -0.131 | -0.4 | 3.059 |  |  |  | 4 |
|  | Z401J | -0.474 | -0.589 | -0.486 | 3.184 |  |  |  | 4 |
|  | Z7W3K | 1.898 | -0.585 | -0.434 | -0.504 |  |  |  | 4 |
|  | Z401D | -0.578 | -0.533 | -0.402 | 2.474 |  |  |  | 4 |
|  | Z9Z0W | -1.059 | -0.585 | 0.023 | -0.585 |  |  |  | 4 |
|  | Z401L | -1.346 | -0.889 | -0.355 | -0.494 |  |  |  | 4 |
|  | Z9Z0Z | -4.25 | -0.889 | -0.464 | -0.454 |  |  |  | 4 |
| MET | Z8I3U | -0.585 | -0.085 | -0.342 |  |  |  |  | 3 |
|  | Z714D | 0.106 | -0.306 | 0.585 |  |  |  |  | 3 |
|  | Z9Z0Y | -0.811 | -0.585 | 2.556 |  |  |  |  | 3 |
|  | Z7692 | 0.446 | -0.68 | -1.059 |  |  |  |  | 3 |
|  | Z6866 | -0.585 | -1.358 | 0.585 |  |  |  |  | 3 |
|  | PubChemCID86554 | -0.318 | -0.336 |  |  |  |  |  | 2 |
|  | PubChemCID52925145 | -0.088 | -0.233 |  |  |  |  |  | 2 |
|  | Z401W | -0.102 | -0.537 |  |  |  |  |  | 2 |
|  | Z57S3 | 0.585 | -0.223 |  |  |  |  |  | 2 |
|  | Z8C3U | -0.51 | -0.585 |  |  |  |  |  | 2 |
|  | Z8I3O | -0.278 | -0.281 |  |  |  |  |  | 2 |
|  | Z944W | -0.128 | -0.464 |  |  |  |  |  | 2 |
|  | Z5J0G | -0.628 | -0.662 |  |  |  |  |  | 2 |
|  | Z4E58 | 0.506 | 0.333 |  |  |  |  |  | 2 |
|  | Z6Q83 | -0.13 | 0.585 |  |  |  |  |  | 2 |
|  | Z7060 | -0.585 | -0.059 |  |  |  |  |  | 2 |
|  | Z4S4R | 0.084 | -0.404 |  |  |  |  |  | 2 |
|  | Z57S1 | 0.585 | -0.585 |  |  |  |  |  | 2 |
|  | Z7059 | -0.059 | -0.585 |  |  |  |  |  | 2 |
|  | Z401V | -0.578 | 0.585 |  |  |  |  |  | 2 |
|  | Z4H33 | -0.585 | 0.837 |  |  |  |  |  | 2 |
|  | Z57H7 | 1.098 | -0.585 |  |  |  |  |  | 2 |
|  | Z5G77 | 1.134 | -0.234 |  |  |  |  |  | 2 |
|  | Z5V4D | 1.416 | 0.585 |  |  |  |  |  | 2 |
|  | Z7W3L | -0.585 | 2.474 |  |  |  |  |  | 2 |
|  | Z6W2S | -0.644 | -1.089 |  |  |  |  |  | 2 |
|  | Z9178 | -0.302 | -1.12 |  |  |  |  |  | 2 |
|  | Z4O47 | -0.224 | -2 |  |  |  |  |  | 2 |
|  | Z4U71 | -1.101 | 0.433 |  |  |  |  |  | 2 |
|  | Z4465 | -1.059 | 0.585 |  |  |  |  |  | 2 |
|  | Z510L | -1.218 | 0.381 |  |  |  |  |  | 2 |
